# Therapeutic Challenges to Retinitis Pigmentosa: From Neuroprotection to Gene Therapy

**DOI:** 10.2174/138920211795860062

**Published:** 2011-06

**Authors:** Jayashree N Sahni, Martina Angi, Cristina Irigoyen, Francesco Semeraro, Mario R Romano, Francesco Parmeggiani

**Affiliations:** 1 St. Paul’s Eye Unit, Royal Liverpool University Hospital, Liverpool, UK; 2 Department of Ophthalmology, University of Brescia, Brescia, Italy; 3 Department of Health Sciences, University of Molise, Campobasso, Italy; 4 Department of Ophthalmology, University of Ferrara, Ferrara, Italy

**Keywords:** Gene therapy, neuroprotection, retinal prosthesis, retinal transplant, retinitis pigmentosa, treatment.

## Abstract

Syndromic retinitis pigmentosa (RP) is the result of several mutations expressed in rod photoreceptors, over 40 of which have so far been identified. Enormous efforts are being made to relate the advances in unraveling the patho-physiological mechanisms to therapeutic approaches in animal models, and eventually in clinical trials on humans. This review summarizes briefly the current clinical management of RP and focuses on the new exciting treatment possibilities. To date, there is no approved therapy able to stop the evolution of RP or restore vision. The current management includes an attempt at slowing down the degenerative process by vitamin supplementation, trying to treat ocular complications and to provide psychological support to blind patients. Novel therapeutic may be tailored dependant on the stage of the disease and can be divided in three groups. In the early stages, when there are surviving photoreceptors, the first approach would be to try to halt the degeneration by correction of the underlying biochemical abnormality in the visual cycle using gene therapy or pharmacological treatment. A second approach aims to cope with photoreceptor cell death using neurotrophic growth factors or anti-apoptotic factors, reducing the production of retino-toxic molecules, and limiting oxidative damage. In advanced stages, when there are few or no functional photoreceptors, strategies that may benefit include retinal transplantation, electronic retinal implants or a newly described optogenetic technique using a light-activated channel to genetically resensitize remnant cone-photoreceptor cells.

## INTRODUCTION

The term retinitis pigmentosa (RP) encompasses a heterogeneous group of disorders that result in an initial loss of rod photoreceptors, followed by a detrimental effect on cone photoreceptors, inner nuclear layers, ganglion cells and retinal pigment epithelium (RPE) [1, 2].

Rods represent 95% of all the photoreceptors in the humans [3] and are responsible for scotopic vision, while cones are responsible for photopic vision, color vision and fine vision and increase in density towards the centre of the macula [4].

The photoreceptors consist of an outer segment (OS), an inner segment, a cell body and a synaptic terminal where neurotransmission occurs to second order neurons. The OS discs are renewed in a circadian rhythm from the base of the photoreceptor OS, the RPE engulfs the distal discs where they undergo degradation by phagocytosis, thus keeping the length of the OS constant [5]. Each OS contains several million visual pigment molecules called opsin and vitamin A chromophore, retinal [6]. Photo-activation results in the isomerisation of 11 cis-retinal to all-trans-retinal. Continued function of the photoreceptors requires the removal of the all-trans-retinal and resupply of the chromophore by the RPE cells [7]. All-trans retinyl esters are the substrate for RPE protein 65 (RPE65). In the rod OS, the transmembrane proteins peripherin/retinal degeneration slow (RDS) [8], retinal outer segment protein 1 (ROM1) [9], and rim protein ABCA4 [10] have been studied in the context of RP. ABCA4 encodes the rod and cone photoreceptor rim protein, which is a member of the adenosine triphosphate (ATP)-binding cassette transporter family [11]. It plays a major role in clearance of all-trans-retinal from the disk membranes after photo-excitation of rhodopsin. The most probable substrate of ABCA4 is N-retinylidene- PE, a product of the reaction of all-trans-retinal with phosphatidylethanolamine. During the process of disk shedding, these compounds, of which the best studied is A2E, accumulate in the cells of RPE, which ultimately leads to RPE cell death and concomitant degeneration of photoreceptors [12].

Non-syndromic RP is the result of several mutations expressed in rod photoreceptors, 44 of which have been identified so far [13] (See http://www.sph.uth.tmc.edu/retnet/sum-dis.htm for updated genes and loci). These include mutations in rhodopsin, enzymes of the phototransduction cascade, structural protein mutations in peripherin/RDS or ABCA4, genes expressed in RPE and coding proteins involved in vitamin A metabolism such as RPE65. These mutations may affect the renewal and shedding of the photoreceptor OS, the visual transduction cycle and/or the vitamin A (retinol) metabolism [4]. The retinal phenotypes associated with ABCA4 depend on the severity of the mutation and the remaining activity of the rim protein; patients with two severe alleles and no rim protein activity may have RP [10].

Enormous efforts are being made to relate the advances in unraveling the patho-physiological mechanisms to therapeutic approaches in animal models, and eventually in clinical trials. This review summarizes briefly the current clinical management of RP and focuses on the new exciting treatment possibilities.

## CURRENT CLINICAL MANAGEMENT

To date, there is no approved therapy able to stop the evolution of RP or restore vision, so the current management aims to slow down the degenerative process, to treat ocular complications and to provide psychological support to blind patients [14].

Vitamin therapy has been suggested for almost twenty years as a possible way to slow down the retinal degenerating process [15]. Berson *et al.* extensively investigated the trophic and anti-oxidants effects of vitamins A and E, respectively, on photoreceptors. Long-term vitamin A supplementation at doses of 15,000 units/day has been suggested to reduce the loss of ERG amplitude, while vitamin E supplementation at 400 units/day was thought to have adverse effects [16]. Further studies from the same group have shown that supplementation with 1200 mg/day of docosahexaenoic acid (DHA) in addition to vitamin A initially slowed down the course of the disease, but this beneficial effect did not last over two years [17]. The most recent study from this group supports the use of 12 mg/day of lutein to slow visual field loss among nonsmoking adults with RP taking vitamin A supplementation [18]. The suggested dietary protocol includes the daily intake of 15,000 units of vitamin A, 12 mg of lutein and 1 to 2 three-oz servings of oily fish per week. However, as wittily argued by Massof and Fishman in the editorial introducing Berson’s work on lutein supplementation, none of the studies on vitamin supplementation convincingly proved to be effective in slowing the rate of progression of RP as the investigators made strong arguments based on secondary analyses of subgroup data and, therefore, do not warrant mandating a change in how patients with RP are treated [19].

Moreover, Vitamin A could be toxic and should be avoided in patients with mutations in ABCA4 gene and women planning to conceive or with severe osteoporosis. Liver enzymes, levels of serum retinol and triglyceride should be regularly checked in all patients taking such supplementation.

The most common ocular complications of RP are: posterior central sub-capsular cataract, which can be successfully treated with phacoemulsification and implantation of intraocular lens [20]; macular edema, which can be treated with oral carbonic anhydrase inhibitors (acetazolamide sodium 250 mg BID) [21] or sustained topical dorzolamide therapy [22], with inconstant results; mild inflammatory reactions, which do not require treatment.

It seems to be appropriate, especially in the early stages of the disease, to suggest that RP patients protect their retinas from excessive light. Experiments on photic damage to the retinas of rats with hereditary retinal dystrophy and some tentative clinical evidence on human patients suggest that, in human RP, one could try to protect the rods from bright light in an attempt to delay the retinal degeneration and to prolong the period of useful vision [23]. Hence the use of low vision aids, especially the use of tinted lenses that filter short wavelength Light, can be of benefit in controlling glare and protecting the retina from photopic damage [24].

In later stages of the disease, low vision aids may also be beneficial in maximizing the limited vision. Professional psychological help as well as supportive patients’ associations are also fundamental when dealing with RP patients at the final, blinding, stage of disease. At the time of diagnosis, patients should be informed of the visual prognosis and orientated towards institutions that help them to rehabilitate and obtain new skills. If left to themselves, RP patients have been reported to have difficulties in adjusting to their visual loss particularly with respect to health-care orientation, vocational environment, social environment, and extended family relationships [25].

## FUTURE TREATMENTS

Multiple factors, genomic, biochemical and cellular responses, reduce the viability of photoreceptors in inherited retinal dystrophies. Novel therapeutic approaches have been tailored dependant on the stage of the disease and can be divided in three groups [26]. In the early stages, when there are surviving photoreceptors, the first approach is to try to halt the degeneration by correction of the underlying biochemical abnormality in the visual cycle using gene therapy or pharmacological treatment. A second approach aims to cope with photoreceptor cell death using neurotrophic growth factors or anti-apoptotic factors, reducing the production of retino-toxic molecules, and limiting oxidative damage. In advanced stages, when there are few or no functional photoreceptors, strategies that may benefit include retinal transplantation, electronic retinal implants or a newly described optogenetic technique using a light-activated channel to genetically resensitize remnant cone-photoreceptor cells.

## GENE THERAPY

Gene therapy works best for diseases resulting from the reduction or absence of a protein with an essential function. Assuming a lack of secondary changes, targeting the gene encoding this protein could result in a therapeutic benefit. The principle of gene therapy is the transfer of a therapeutic gene by use of viral or non-viral vectors and requires genetic modification of the ocular cells to produce its therapeutic effect. Gene therapy strategies however differ depending on if the exact gene and type of mutation is known, thus the importance of efficient genotyping.

Methods suggested to replace or correct abnormal genes can classified into two groups: (1) gene augmentation therapies, where a normal gene is inserted into the genome to replace nonviable or diseased genes using a carrier vector, (2) gene silencing therapies, in which the expression of the mutated gene is inhibited by use of ribozyme or RNA interference. 

### Gene Augmentation

In autosomal recessive and X-linked RP, mutations usually lead to a loss of function and the principle of gene therapy is to introduce a wild-type version of the mutated gene into the cells in which normal functioning of this gene is required [27]. Successful gene therapy is dependent on efficient transduction of the target cell and sustained expression of the recombinant virus at a sufficient level. Adeno-associated virus (AAV), a nonpathogenic parvovirus, has been the most successful vector owing to its ability to transduce a variety of non-dividing cell types [28, 29]. RPE cells take up and express recombinant viruses at high efficiency, which represents an important advantage of this approach. Moreover, the immune-privileged status of the eye prevents problems with undesirable immune response to AAV. 

This therapy has been most extensively studied in Leber’s congenital amaurosis (LCA) with mutation in RPE65, which interrupts function of the visual cycle [30]. Preliminary trials involving surgical delivery of recombinant AAV carrying wild type rpe65 into the subretinal space of the Briard dog, a natural occurring model with mutation in the RPE65 gene, demonstrated dramatic improvements in the light sensitivity of rods and cones [31-35]. Importantly, expression of Rpe65 and restoration of vision in the treated dogs was stable over the four-year study period [36]. More than 50 dogs have been treated using this approach, with 95% showing restored vision [37]. The availability of animal models of this condition has enabled preclinical studies of safety and efficacy [38]. Three human clinical trials using AAV serotype 2 reported their findings separately in 9 patients in 2008 [39-41]. To date, over 30 patients have so far received the gene therapy, ranging in follow up from 90 days to 1.5 years. No major side effects have been reported [42,43]. Most patients have shown a sustained improvement in subjective and objective measurements of vision (i.e., dark adaptometry, pupillometry, electroretinography, nystagmus, and ambulatory behavior). The greatest improvement was noted in children, all of who gained ambulatory vision, suggesting that early intervention would result in the best potential gain [44].

### Combined Gene-Silencing and Gene Replacement

Autosomal dominant conditions are mainly caused by toxic gain of function mutations that result in either a mutated protein or a negative effect of the encoded protein. Mutations within the rhodopsin gene account for approximately 25% of the cases of autosomal dominant RP, the rest are not linked to rhodopsin. In RP the aberrant gene product is detrimental to the photoreceptors and ultimately results in cell death. Two approaches have been proposed to silence the abnormal gene: ribozymes and RNA interference (siRNA).

Ribozymes can be designed to cleave mutant mRNA molecules so that the mutant rhodopsin is not produced [45]. The use of ribozyme therapy is however limited by being mutation dependent. This would mean, in the case of rhodopsin alone, targeting more than 100 different alleles.

siRNA, on the other hand, is a mutation independent technique for posttranslational gene silencing [46]. Complete down-regulation of endogenous rhodopsin can be achieved using this method. The second part of this therapy involves the introduction of a codon modified rhodopsin cDNA that is not sensitive to the siRNA interference *via *AAV serotype 5 vector mediated gene transfer [47, 48]. The safety of this technique has yet to be established in large animal and human experiments.

## PHARMACOLOGICAL TREATMENT

Pharmacological agents can also compensate for a biochemical defect and can be a good choice in those cases where some aspects of the patho-physiological mechanism are known. The main advantage of such treatment is that the eventual toxicity of the drug is known and can be modulated. This strategy may be applicable to RP with a primary deficit in chromophore biosynthesis causing an alteration of the visual cycle. Examples include LCA owing to mutations in the LRAT and RPE65 genes.

The first experiments aimed at bypassing the biochemical defect caused by the absence of RPE65 were performed by oral administration of 9-cis-retinyl acetate in RPE65-deficient mice [49, 50]. Electroretinogram (ERG) recordings documented restored light sensitivity, increasing with the number of doses and sustained up to six months after treatment. Similar recovery of visual function was observed following intra-peritoneal injection of 11-cis-RAL into RPE65-deficient mice [51].

High doses of retinoids have been shown to be toxic in numerous studies, including teratogenic effects. However, acute and prolonged treatment of mice with 9-cis-retinoids did not cause obvious adverse effects, raising the hope for a trial of chromophore supplementation in humans [30].

## NEUROPROTECTION

RP is a genetically heterogeneous disease where only 60% of the mutations are known [52]. Genetic strategies are dependent on the identification of the gene mutation, and are therefore restricted in their application. Neuroprotection is a therapeutic strategy for the treatment of retinal neurodegenerative disease that is independent of the etiology of the degeneration. The aim is to provide a protective environment to prolong the viability of the photoreceptors by their effect on the secondary biochemical pathways. This can be achieved either by delivering neurotrophic growth factors, or inhibiting pro-apoptotic pathways, or implementing viability factors such as the rod-derived cone viability factor (RdCVF).

### Neurotrophic Factors

There have been a number of examples of neurotrophic factors being successfully tested in animal models for neurodegenerative diseases, but failing to bring any benefit over placebo when tested in human clinical trials, such as glial cell-derived neurotrophic factor (GDNF) for Parkinson disease [53] or ciliary neurotrophic factor (CNTF) for amyotrophic lateral sclerosis [54]. These experiences show that the efficacy of neurotrophic factors in treating human disease remains to be established, and the delivery of these factors is problematic as they have a short half-life [55].

A number of agents that slow photoreceptor death in animal models have been identified: basic fibroblast-derived growth factor (bFGF), brain-derived neurotrophic factor (BDNF), cardiotrophin-1, nerve growth factor (NGF), fibroblast growth factor (FGF) and CNTF.

Faktorovich *et al.* demonstrated the neuroprotective effect of bFGF in 1990 [56]. Since iterative intravitreal injections are not recommended, other strategies have been tried, such as the use of encapsulated cells producing bFGF placed in the vitreous cavity [57]. However, the occurrence of major side effects such as retinal revascularization has excluded its utility in a clinical setting.

The use of encapsulated cells secreting CNTF into the vitreous has been extensively studied and has shown preservation of retinal integrity in different animal models [58]. A phase I safety trial on 10 participants over a period of six months using an intravitreal implant device (NT-501, Neurotech USA) was well tolerated [59]. A positive trend in visual acuity was also noted, however ERG responses were inconsistent. A phase III clinical trial is currently ongoing to investigate whether CNTF can improve photoreceptor function, in terms of visual acuity and visual field sensitivity, in RP patients [60].

### Inhibitors of Apoptosis 

A final common pathway of all types of RP is photoreceptor cell death [61]. Leonard *et al*. reported their non-specific approach targeting the apoptotic pathway involving a family of cysteine proteases known as caspases [62]. The X-linked inhibitor of apoptosis (XIAP) protein is thought to be the most potent member and this group employed AAV mediated delivery of XIAP to study its neuroprotective effect. XIAP treated eyes of homozygous albino transgenic rats had significantly preserved outer nuclear layer than their contra-lateral untreated counterparts. The authors believe that this technique may be more widely applicable as it is independent of mutation.

### Calpain Inhibitors

Recent evidence derived from studies on retinal degeneration (rd1) human homologous mouse model for RP suggests that photoreceptor cell death may result primarily from non-apoptotic mechanisms, where photoreceptor cell death appears to be governed by, among other things, changes in cyclic nucleotide metabolism, down-regulation of the transcription factor CREB, and excessive activation of calpain and PARP [63]. Pharmacological inhibition of calpain activity in rd1 organotypic retinal explants using the highly specific calpain inhibitor calpastatin peptide reduced photoreceptor cell death *in vitro* after both short and prolonged exposure, an effect that was also evident after *in vivo* application *via *intravitreal injection. These findings highlight the importance of calpain activation for photoreceptor cell death but also for photoreceptor survival and propose the use of highly specific calpain inhibitors to prevent or delay RP [64].

### Rod-Derived Cone Viability Factor

Cone death in RP is also caused by lack of surviving factors released by the normal retina. This has been confirmed by the discovery that rods generate a diffusible factors stimulating cone survival in the retinal degeneration mouse model [65]. One of the trophic factors has been identified by expression cloning and named rod-derived cone viability factor (RdCVF) [66]. RdCVF protein injections in a frequent type of rhodopsin mutation, the P23H rat, induced an increase in cone cell number and, more important, a further increase in the corresponding ERG, making it a promising therapeutic option [67].

## ELECTRONIC RETINAL IMPLANTS

Gene therapy and neuroprotective agents play a role in rescuing and preventing the loss of photoreceptors. However, in advanced stages of the disease, where the loss of photoreceptors is already established, and for the majority of patients in which the mutation is not known, the electronic implants or artificial retina can be an option for restoration of vision.

One of the best examples of electronic devices used in medicine are the cochlear implants to treat deafness [68]. In the eye, the electronic devices are meant to replace dead or degenerated photoreceptor cells. These devices capture images and convert them into an electronic signal. The electronic signal is sent to the retinal secondary neurons (ganglion cells, bipolar cells, etc.) that transmit it to the visual cortex *via *the optic nerve and pathway. Hence, these devices must be able to connect with viable secondary neurons. Several studies have demonstrated the viability of these secondary neurons in patients with RP [69]. Optical coherence tomography (OCT) has been used as a objective non-invasive method to assess the presence of viable inner retinal neurons (secondary neurons) [70] and study the tolerability and long-term effects on the retina of electronic prosthesis.

There are four main types of electronic implants developed so far: cortical visual prosthesis, supra-choroidal implant, epiretinal implant and subretinal implants.

### Cortical Visual Prosthesis

It is based on direct stimulation of the visual cortex bypassing the eye. A group from the Illinois Institute of Technology is planning the first human intra-cortical visual prosthesis [71].

### Supra-Choroidal Implant

Tokuda *et al.* have implanted on rabbit retina a multi-chip flexible stimulator in the supra-choroidal sclera, successfully obtaining Electrically Evoked Potential on visual cortex evoked by the multi-chip stimulator [72].

### Epiretinal Implant

The implant is tacked to the surface of the inner retina. Three groups are involved with this type of implant: the Learning Retina Implant developed by Feucht *et al. *[73], the EPI-RET project that uses an intraocular lens (IOL)-type receiver unit [74], and the group at the Doheny Eye Institute, USC Medical School, in conjunction with Second Sight Medical Products (SSMP). The latter, known as the Argus II retinal prosthesis system, consists of a surgically implanted 60-electrode stimulating microelectrode array. Recently, in a study on the largest cohort of visual prosthesis recipients to date (27 patients), they found that artificial vision augments information from existing vision in a spatial-motor task [75].

### Subretinal Implant

The implant is placed subretinal above the RPE. Theoretically, this is the most physiological placement, as it is placed were the damaged photoreceptors are located. Zrenner *et al.* from the University of Tubingen, Germany, have implanted a subretinal prosthesis in humans and have detected restoration of photoreceptor function *via *electronic devices electrically coupled to inner retinal neurons [76]. The implant, placed under the macular region, contains an array of 1500 active micro-photodiodes (‘chip’). Three previously blind patients with RP could locate bright objects on a dark table; two of them differentiated correctly the direction of fine stripe-patterns. One patient also spontaneously reported new objects with typical shapes such as a banana. He was able to discern shades of grey with only 15% contrast, to localize and approach a person freely, to read the hands of a clock face, and to read letters of the alphabet at room light and combine them to words, even to the point of spontaneously identifying a deliberate spelling mistake.

Another project based on the subretinal implant is the Boston Retinal Implant Project developed by Rizzo *et al.* [77]. They have assessed the concordance of the form of induced perception and the pattern of electrical stimulation of the retina, and the reproducibility of the responses in five patients affected with severe RP.

Several clinical studies have demonstrated the tolerance of the implanted devices, with the exception of the EPI-RET with a large IOL-type receiver unit that caused retinal disruption in a rabbit model [74]. However, the visual benefit is not straight forward, as even limited sight restoration is a slow, learning process that takes months for improvement to become evident [78]. The learning curve could be expected to be easier in younger patients and earlier stages of the disease, due to neuronal plasticity.

In order to properly assess the performance of these artificial retinas there is a need for development of standardized visual tests. These should be able to detect small improvements in vision in patients with advanced RP and capture aspects that relate to a better quality of life [78].

Devices with new, sophisticated designs and increasing numbers of electrodes as well the possibility of using adjunctive therapy, such as neurotrophic agents [79], could allow for long-term restoration of functional sight in patients with improvement in object recognition, mobility, independent living, and general quality of life.

## ARCHAEBACTERIAL HALORODOPSIN

A novel mechanism to create electric signals in the visual pathway that substitute for the usual input from photoreceptors, besides for electronic retinal implants, is the use of a light-activated channel.

In most retinal degenerations, photoreceptor degeneration precedes inner retinal degeneration by several years. It has been suggested introduction of bacterial rhodopsin through gene transfer can render the cells of inner retina photosensitive, thus substituting for the usual input from photoreceptors. There was a restoration of basic vision driven behavior in the rodent. Expression of the bacterial protein channelrhodopsin-2 would generate a light-gated cation channel within the cellular membrane that would allow for depolarization events upon light stimuli [80].

In RP, cone photoreceptors survive longer and may be accessible to therapeutic manipulation. Busskamp and colleagues reactivated the remnant cones in two mouse models of RP by the introduction of a bacterial form of rhodopsin, halorhodopsin, through AAV mediated gene transfer [81]. The reactivated cones enabled RD mice to perform visually guided behaviors.

## RETINAL TRANSPLANT

Retinal transplant is another therapeutic strategy to restore vision in patients with advanced degenerative retinal disease and it is an area of increasing interest in retinal research. The same principle of retinal prosthesis can be applied to retinal transplant, but in this case new cells instead of electronic devices replace the degenerated photoreceptors. The retina is a good substrate for stem cell therapies, as it has the optimal combination of ease of surgical access, combined with the possibility to assess transplanted cells directly through the clear ocular media [82].

Different tissues can be source of cells that can be used for transplantation: fetal tissue, embryonic stem cells, neural stem cells, somatic cells, induced pluripotent stem cells and RPE.

### Fetal Tissue

Studies using whole sheets of fetal neural retina transplanted into the subretinal space have shown survival and differentiation of the grafted tissue [83, 84]. Some studies have described improvements in vision although it is unclear whether it is just due to enhanced survival of host photoreceptors *via *trophic signals from the donor tissue [85-87].

The main limitation of this type of transplant is physical because when transplanting the whole neural retina there is a problem with space and disorganization of host neural retina. It would be more physiological to try to transplant only a sheet of immature photoreceptors [88].

### Stem Cells

Stem cells that could be used for photoreceptor replacement are embryonic stem cells (ESC) and neural stem cells (NSC) isolated from the adult mammalian brain and the adult ciliary margin of the mouse and human eye [89]. Limitations include the ethical problems when working with embryonic stem cells and how to direct the correct differentiation into specific adult cell types with their proper function [88].

### Therapeutic Cloning

Also known as somatic cell nuclear transfer (SCNT). The basis of this technique is to create embryonic stem cells that could differentiate into photoreceptors from an adult somatic cell of the patient [88]. One of the advantages of SCNT is that the risk of immunological rejection is alleviated because the patient's own genetic material is used. Although research in this field is progressing rapidly, there are no results applicable to retinal dystrophies as yet.

### Induced Pluripotent Stem Cells (iPSC)

iPSC are a type of pluripotent stem cell artificially derived from a non-pluripotent cell, typically an adult somatic cell, by forcing the expression of specific genes. Depending on the methods used, reprogramming of adult cells to obtain iPSC may pose significant risks that could limit its use in humans. For example, if viruses are used to genomically alter the cells, the expression of oncogenes may potentially be triggered [90]. The method of reprogramming has been optimized to avoid the use of retroviruses, making the process considerably safer [82]. Recent development of human iPSC and the ability to specifically induce differentiation into cells with human photoreceptor phenotype has now provided us with the opportunity for embryo-free autologous transplantation and opens a new path in retinal transplant [91].

### RPE Transplantation

The RPE constitutes with the photoreceptor layer a functional unit that provides the transducing interface for visual perception [7] and it also plays an important role in the maintenance of the extracellular matrix. These properties have encouraged many researchers to try to improve vision by RPE transplantation. Radtke *et al.* published a series of 10 patients affected by RP and dry age-related macular degeneration (AMD) who received implants of neural retinal progenitor cell layers with RPE [92]. The study showed improvement in VA in 70% of patients and demonstrated the efficacy and safety of the RPE transplant in animal models of retinal degeneration. One disadvantage of this transplantation technique is that it will be of limited benefit in patients with severe loss of photoreceptors [93]. The hypothetical combination of RPE transplantation with other modalities of treatment, such as gene transfer, remains an exciting future prospect [94].

The treatment of RP patients by photoreceptor precursor cell transplantation to the human retina remains a promising strategy for retinal repair. For retinal dystrophies caused by photoreceptor-specific gene mutations, autologous adult derived cells do not initially appear to be the best source of new retinal neurons, as the genetic mutation will remain. Future treatment for retinal degeneration due to photoreceptor cell loss may require a combination of gene and cell therapeutic strategies [39, 95].

## CONCLUSIONS AND CHALLENGES

The future for treating this group of devastating inherited diseases has never looked brighter. Approaches to therapy for RP now include: gene therapy, neurotrophic growth factors, anti-apoptotic agents, ribozyme therapy, RNAi, retinal transplantation, dietary supplementation, retinal prostheses, and stem cell therapy [96].

However, there remain several hurdles:

The success of these treatments depends on proper patient selection, which may include the stage of the disease, the patient’s age, systemic and environmental factors and well set out outcome measures of improvement.

Successful translation of new therapies also requires animal models of the disease. There are different natural and transgenic RP animal models that have different times to developing photoreceptor degeneration. It may be difficult to decide which model is most relevant to a specific study. Some of the mutations between humans and animals are not similar. The mutation and the phenotype in the animal model must be viewed with some degree of caution; it cannot be assumed that these are truly representative of the disease that is occurring in the patient until the phenotypes are critically examined using the same criteria.

There is tremendous genetic heterogeneity associated with the RP phenotype and accurate genetic characterization is essential for specific gene therapies. It is not known whether partial reversal of the biochemical defect will change the course of progressive retinal degeneration. Although RPE65-LCA has been extensively studied, a predictive relation between a pair of mutant RPE65 alleles and resulting disease severity is currently unknown. There may be considerable intraretinal variation of retinal dysfunction, degeneration and RPE health. Better understanding of the contribution of specific genotypes to disease severity may allow such predictions in the future.

Human results suggest that there may even be differences between foveal and extra-foveal cones and this may explain the lack of foveal functional improvement demonstrable with gene therapy to date [97]. Thus treating the fovea may not lead to fovea-specific increases in visual acuity, but this rather important issue has still not been resolved.

The importance of the RPE has not yet been established. In late stages, with evidence of diffuse disease of the retinal pigment epithelium, the genes that are transfected into the photoreceptor cells may not be sustainable by the RPE. Some patients have changes not only in the RPE, but also in the choroidal circulation.

Another issue is the variation in clinical presentation of patients with RP. It will be important for clinical trials in RP to randomize patients along the lines of recognizable phenotypes, because there could be a different response to various therapeutic procedures among the various phenotypes.

So far gene therapy trials have involved patients with almost no vision. The next daunting step is to include patients with expanses of useful and sometimes only moderately abnormal vision. High expectations for remarkable visual recoveries may have to be replaced by the less exciting but still useful outcome of slowed progression of these ultimately blinding disorders.

Effective delivery of the therapy, both genetic material and neurotrophic factors to the target tissue has been a formidable task. The blood-retinal barrier prevents most drugs administered topically or systemically from reaching an effective dose in the retina. One of the key methods would involve methods to introduce the molecule without causing a retinal detachment. This has been achieved to some extent with CNTF encapsulated cell technology, thereby achieving adequate intraocular concentration of the drug and limiting systemic toxicity. However, weekly, monthly or yearly intraocular injections represent distinct disadvantages to patients who require treatment during a lifetime. Longer follow up is needed to establish the success of CNTF technology, which may then be applicable for other treatment types.

Translational clinical research initiatives are finally offering hope to relatives and patients with RP. Genotyping is one of the biggest resource challenges we face. State- of-the-art clinical facilities are needed to administer and evaluate novel investigational therapies. Even if the promise of gene replacement therapy is fully realized, the basic research and clinical trials that precede successful gene therapy take years. We suggest that research should be directed to identifying the patho-physiological processes common to all the photoreceptor degenerations and a more general treatment would fill an enormous therapeutic gap.
